# Recent developments, challenges and opportunities in genome editing for crop science from a societal perspective

**DOI:** 10.3389/fgeed.2025.1568072

**Published:** 2025-06-03

**Authors:** Srividhya Venkataraman, David Zaruk, Kathleen Hefferon

**Affiliations:** ^1^ Department of Cell and Systems Biology, University of Toronto, Toronto, ON, Canada; ^2^ Retired, Brussels, Brussels, Belgium; ^3^ Department of Microbiology, Cornell University, Ithaca, NY, United States

**Keywords:** genome editing, agriculture, consumer acceptance, risk assessment, off target effects

## Abstract

Genome editing has presented enormous potential in the fields of medicine and agriculture. Here, we explore the social and regulatory aspects of genome editing from the perspective of food security. We provide recent examples of crop genome editing successes. We discuss the current regulatory framework for genome edited crops in North America and Europe, and present how public perception can influence international policies and trade.

## Introduction

Genome editing has hit the world by storm, and while its applications for the field of medicine are reflected in the news each week, its potential in agriculture cannot be overlooked. Genome editing is a technology which can change the hereditary blueprint, or DNA, of any organism in a highly specific manner by as little as an insertion or deletion of a single nucleotide, so that minor changes in gene expression can be realized (site directed nuclease −1 or 2 (SDN-1 or SDN-2) events), or by the addition of entire genes (SDN-3), at a designated target site within the genome. It is this specificity and ease of use that differentiates genome editing from other forms of genetic engineering, such as transgenesis or the generation of transgenic plants.

This review describes the benefits of genome editing in agriculture and provides examples of companies, various organizations and universities using this technology for current crop development. Representatives of several genome-edited crops with novel characteristics are presented. Disadvantages of the genome editing technology such as off-target effects, long-term effects and other concerns are discussed. The review then turns to the regulation of genome edited crops in North America, as well as regulatory obstacles in Europe. Challenges and opportunities in crop science genome editing from a societal perspective are discussed. The review concludes with prospects for genome editing for agriculture in the future.

### Recent developments regarding genome editing in crops

Increasingly, plant breeders are adopting genome editing technology as a quick, precise, efficient and affordable method of modifying crops. Moreover, plant scientists can apply this knowledge to select useful genes in crops such as corn, canola and wheat for gene editing towards developing novel, enhanced varieties. Using gene editing, genes already occurring in the plant can be switched on and switched off as desired. This would be beneficial for agriculture as it would mean the rapid availability of novel crop varieties with low seed prices. A market rush has started for gene edited crops, as regions and nations brace themselves to participate in potential enhancements in crop performance and profits.

The number of examples of companies who leverage genome editing for crop development is increasing. For example, to further its belief in the immense potential of cutting-edge genome editing technologies to address the world’s prevalent nutritional problems, the agribusiness giant Bayer has disclosed features of a novel open innovation manifesto in addition to state-of-the-art collaborations to generate new varieties of tomatoes to address vitamin D deficiency and develop genome edited varieties of leafy greens. Bayer has launched two initiatives with external collaborators to produce genome edited vegetables. Bayer along with G+FLAS, a South Korean biotech company have involved in an agreement towards collaborating on the development of genome-edited varieties of tomato biofortified with vitamin D3 (Morrison, 2024). On a global scale, the occurrence of vitamin D deficiency is quite common, especially in countries with limited sunlight during the winter season. Worldwide, it is estimated that at least a billion people are affected by vitamin D deficiency which can result in several health problems, including rickets. This collaboration harnesses the genome-editing technology of G+FLAS as well as the tomato germplasm proprietary to Bayer. This technology alters the plant genome similar to that occurring in nature or via conventional breeding, but in a quicker and more precise manner.

The significance of Bayer’s judicious open innovation strategy has been proven recently by means of a new licensing accord with USA-based pairwise, a trailblazing food and agritech startup located in North Carolina. In 2023, Bayer had obtained license authorization from Pairwise, that provides rights to manipulate and commercialize mustard greens genome edited by Pairwise (Mullin, 2024). Pairwise used CRISPR-based editing to remove many copies of the gene that confers pungency while retaining antioxidants, fiber content as well as other nutrients present in mustard greens. These greens are colorful, possess fresh flavor and are nutritionally enhanced. They are the first food to be introduced into the market in North America following genome editing. This provides a great-tasting, more appetizing new salad addition with higher nutrient content. This recent deal generates value beyond merely the sale of the product, as it is accompanied by rights to apply the knowhow, technology and intellectual property proceeding ahead. However, even as many years of research have determined that genetically altered crops are healthy and safe just as their conventionally bred equivalents, public misperception about how GMOs are generated, and their ostensible risks prevail. Both Pairwise and Bayer hope that this introduction of gene edited mustard greens would induce consumers to be more welcome to genome-edited foods in contrast to conventional GMOs that have encountered backlash from consumers in the United States and other countries.

Genetic engineering of crops to make them colorful could aid farmers to generate foods without applying herbicides, since this would enable them to spot weeds more easily (Horton, 2024). The genomes of crops could be changed so that they produce pigments such as anthocyanins that provide carrots their orange color and blueberries their blue color. The plant *Chenopodium album* is cultivated for its nutritious seeds in Nepal and India in addition to having been used as a food source in European countries in the iron era. In the present time, this plant commonly called fat hen, is a competitive, strong weed growing in fields of Europe that incurs notable crop losses. Scientists are contemplating improvement of fat hen to render it to be a novel sustainable crop that dispenses with the need for much care in order to cultivate it. If this materializes, then distinction of the improved version of fat hen from its weedy, wild counterpart could make its cultivation more facile, as it would enable easier weeding without the use of herbicides. Such plants that are visually distinctive could facilitate robot weeders to differentiate with ease between the desired version of such crops from weeds. Therefore, utilization of genome editing to promote their recognition in a visually distinctive manner that aids weeding robots to easily cull unwanted weeds could efficiently address this matter. Common crops such as maize and wheat can be gene edited to make them brightly colored for easy distinction by weeding robots, so as to reduce the requirement for herbicides. Crops could be cultivated to grow leaves of unusual shapes or have properties that are detectable through sensors while being indiscernible to the naked eye such as the ability to emit in the infrared spectrum. This kind of new domestication can generate crops having better yields and more environmental sustainability in addition to having the capability to be distinguished from their wild, ancestral counterparts. This means that crop genomes can be edited to include genes encoding pigments sourced from other plants.

The start-up company, Sanatech Seed Co., Ltd., is collaborating with University of Tsukuba, Japan to develop the “Sicilian Rouge High GABA” F1 variety having augmented GABA content acquired using genome editing (Ezura, 2022). Pioneer EcoScience is involved in the commercialization and marketing of this product in addition to generating more new varieties that meets the necessities of both growers and consumers. GABA is considered as a functional constituent in foods due to its capability to decrease blood pressure and induce requiescence in humans (Yoshimura et al., 2010). In natural conditions, tomato contains high GABA levels. Nevertheless, to acquire functional outcomes with existing tomato varieties, it is essential to consume high amounts of tomatoes. Investigators identified the SlGAD3 gene as being critical for the accumulation of GABA in fruits (Takayama et al., 2015). Further, they deleted the SlGAD3 autoinhibitory domain (AID) which led to the enhancement of GABA accretion particularly in fruits (Takayama et al., 2017). They introduced a stop codon mutation preceding the nucleotide sequence that encodes AID of the endogenous SlGAD3 using the CRISPR/Cas9 technology which led to high concentrations of GABA in these tomatoes. Subsequent to the establishment of high accretion GABA technology in the experimental varieties of tomatoes, Sanatech Seed Co. Ltd. was formed in 2018 (Ezura, 2022) to serve as a venture to bolster its social execution, whereupon it acquired commercial license for the application of the CRISPR/Cas9 technology. In actuality, the company’s commercial variety of tomato, “Sicilian Rouge” was genetically modified to accrue high GABA levels. The company now delivers these GABA-enriched tomatoes to consumers directly. This launch of the first genome edited tomatoes in Japan, is expected to have major impact on the advancement trend of genetically engineered crops throughout the world.

Pairwise is also involved in developing 16 crops thus far using gene editing technology (Greig, 2024). The logic behind this pursuit is that enhancement of vegetables and fruits would invite people to eat more of such edited crops. Pairwise is generating corn that has higher yield reaching as high as a 10% enhancement after just 2 years of its development. Additionally, a seed company in Germany, the KWS Group is also contemplating the use of gene editing technology towards developing crops such as sugar beets, sunflower, corn and cereals having resistance to pests, viruses and fungi. Enhancement of crops such as pulses and flax through gene editing is being considered by some companies based in Canada. In Africa, gene edited sorghum, teff and corn are nearing release for consumption. As announced by the Agriculture and Agri-Food Canada, John Laurie, a researcher from the Lethbridge Research and Development Center is developing a gene edited new wheat variety that is capable of resisting drought (Greig, 2024). In the USA, a bioventure company called Calyxt generated a soybean line with high oleic acid in its oil called Calyno by means of TALEN technology (Transcription Activator-Like Effector Nuclease) (Ezura, 2022) (Pearce, 2024) while Corteva is generating waxy corn using gene editing technology.

Thus far, 23 crops including soybean, wheat, rice, potato and corn have been enhanced using the genome editing technology (Jenkins et al., 2021). The Cross-Ministerial Strategic Innovation Promotion Program formulated in Japan has been executing the advancements of agricultural produce using the genome editing technology. Besides the high-accumulation GABA tomato lines, rice having augmented yield, wheat with repressed sprouting at the preharvest stage and potatoes having suppressed production of natural toxin have also been generated. Of these, the latter two are candidate genome edited crops for commercialization in the near future for which field trials began in 2021 (Ezura, 2022). Moreover, several other crops are being bred using this technology. Japan was the first nation in the world to introduce an unprocessed genome edited crop into the market and presently leads the world in social application of this technology. The fruitful launch of high GABA tomatoes has had a significant impact on advancing the rules of handling such GE crops on a global scale. Therefore, genome editing is a propitious futuristic technology that would pave the way for rapid progress and launch of quantified and qualified crops that would promote sustainable food production worldwide.

In a pioneering venture, the independent biotechnology company Hudson River Biotechnology (HRB), established in Wageningen, Netherlands, proclaimed a milestone accomplishment wherein strawberry plants were successfully regenerated from single, gene-edited cells using TiGER, its proprietary CRISPR system (Sanchez, 2024). Increasingly, the worldwide berry sector is beginning to focus on quality. Conventionally, strawberry fruit breeding for disease resistance and quality enhancement has been a protracted process owing to the genetic complexity of this plant. Strawberries contain 8 sets of chromosomes which significantly complicates traditional breeding protocols. The technology of gene editing affords a propitious solution to rapidly introduce favorable genetic traits in this fruit. Using the TiGER workflow, HRB produces novel plant varieties starting from a gene edited single cell including automated screening for several conditions of regeneration to select the appropriate combination of processes for each variety/crop. This has been found to be an effectual, scalable strategy for accelerating the introduction of favorable traits for various crops in the market. The TiGER technology has therefore unlocked the potential for augmentation of fruit taste, flavor, sustainability and nutritional value in the notably recalcitrant strawberry crop.

The CRISPR/Cas9-edited Golden Promise variety of barley is currently under field trial in Switzerland wherein the CKX2 gene involved in the regulation of seed formation has been disabled. Such a strategy has already been used successfully to augment the yield of rice plants. Researchers at Swiss agricultural institute Agroscope along with their partner Freie Universität Berlin, hope to achieve similar results in barley (Hunt, 2023). The following Table 1 highlights some examples of GE crops developed by universities and major biotech companies.

**TABLE 1:** Examples of gene edited/NBT (New Breeding Techniques) crops successful in laboratory studies or are in field trials or approved for sale by biotechnology companies (sources (Project, 2024): https://crispr-gene-editing-regs-tracker.geneticliteracyproject.org/canada-crops-food/#:∼:text=ApprovedEasilydigestiblecorn%2C2020,tobeabsorbedmorequickly) (Chapparro, 2024).

Product	Description	Country	Company/University	Reference
Low acrylamide potato	CRISPR used to disrupt the genes responsible for the synthesis of precursors of acrylamide	Australia	Murdoch University, Western Australia	Ly et al. (2023)
Seedless blackberries	Gene editing used to develop seedless, thornless and compact blackberries	United States of America Entered field trials 2024	Pairwise	Barefoot (2024b)
Non-browning avocado	Used CRISPR to disrupt polyphenol oxidase production, crucial to the browning process	United States	GreenVenus	LLC (2023)
Vitamin D tomato	Performed CRISPR editing to accumulate provitamin D3 in the tomato fruit	UNited Kingdom	John Innes Center	Li et al. (2022a)
Sorghum witchweed resistant variety	CRISPR used to introduce edits to generate witchweed resistance	Kenya, Africa	Kenyatta University, Nairobi	Ledford (2024)
Cowpeas suitable for mechanical harvest by synchronized flowering	CRISPR editing targeted genes responsible for plant architecture and flowering time	Israel 2023	Better Seeds	Achard (2023)
Pennycress with reduced erucic acid content and premature seed shattering	Knocked out genes essential for the accretion of erucic acid and seed shattering	United States	CoverCress	Chapparro (2024)
Waxy corn	Corn with high starch content developed using CRISPR	Approved: Japan (2024)	Corteva Agriscience	Chilcoat (2020)
Poplar trees with enhanced wood fiber content	CRISPR targeted lignin biosynthesis and other woody carbohydrates	United States	North Carolina State University	Kulikowski (2023)
Cannabis variety more friendly to the industry	Knockout of the CBDAS gene necessary for the biosynthesis of CBD (cannabidiol) and THC (delta-9-tetrahydrocannabinol)	USA	University of Wisconsin	Adlin (2024)
Fungal resistant grape variety	Edit to improve resistance to powdery mildew	United States	VitisGen	ISAAA (2023)
Sugarcane with improved biomass	Edits to change the angle at which leaves emerge from the primary stem, thus increasing the efficiency of light capture	United States	University of Florida	News (2024)
Fungal resistant wheat	Edit approved that confer resistance to a common fungal infection called powdery mildew that can be applied to different varieties	Approved: China (2024)	Suzhou, Chinese Academy of Sciences	Li et al. (2022b)
Non-browning lettuce	GreenVenus Non-browning romaine lettuce	Approved: United States (2024)	Intrexon	US (2019)
Non-browning banana	Banana developed using CRISPR to slow the browning process for prolonged shelf-life	Approved: Philippines (2023)	Tropic Biosciences	Devlin (2025)
Non-browning mushroom	White Button Mushroom Non-browning mushroom developed using a gene-editing technique called TALENs	Approved: United States (2016)	Pennsylvania State University	Waltz (2016)
Non-browning potato	White Russet Potato Non-browning, blight protection, lowered sugars, and low acrylamide potato developed with RNA interference	Approved, available: United States (2015) Available: Canada (2015)	Simplot	Canada (2017)
Mustard greens	Conscious Greens Milder, less bitter mustard green developed using CRISPR-Cas12a	Approved, available: United States (2023)	Pairwise	Barefoot (2024a)
GABA tomato	Sicilian Rouge Tomato edited using CRISPR to contain more GABA, a compound in tomato fruits and known to lower blood pressure	Approved, available: Japan (2021)	Sanatech Seed	Waltz (2022)
High-oleic soybean oil	Calyno Soybean oil with fewer saturated fats and zero trans fats, developed using a gene-editing technique called TALENs	Approved, available: United States (2019)	Calyxt	Tome (2021)
Non-browning apple	Arctic Apple Non-browning apple (multiple varieties) developed with RNA interference, a more traditional New Breeding Technique (NBT). Varieties include Golden, Granny, Fuji, Gala, Honey	Approved, available: Canada (2017) Approved, available: United States (2015)	Okanagan Specialty Fruits	Fruits (2025)

Equipped with predictive design tools that are powered by multiplex gene editing technologies and artificial intelligence, scientists at Inari are currently working on producing enhanced varieties of wheat, corn and soybean meant for commercial use (Thomas, 2025). Inari has generated an AI-based technology to recognize gene sequences that are responsible for plant performance towards editing these traits and boosting desirable characteristics. In addition, their multiplex editing system enables the simultaneous editing of several genes. Traits targeted include enhanced plant yield, and the improved capability of plants to grow under less nitrogen and less water.

Elo Life Systems has used gene editing of vegetables and fruits towards obviating the extinction of various varieties due to factors such as climate changes. Banana varieties with resistance to the deadly fungus Tropical race 4 (TR4) are currently being developed by Elo Life Sciences using gene editing technology. Here, they have performed only small alterations to the genome of the Cavendish banana cultivar that is naturally resistant to TR4 towards strengthening its fungal resistance characteristics. These gene-edited bananas are presently grown in greenhouses and are being tested with large TR4 inoculations to validate their editing strategy. Now, farms in Latin America are involved in conducting field trials of these edited bananas.

Kale lacking bitter aftertaste, bananas resistant to viruses and drought-resistant rice are all moving towards fields and the market at a breakneck pace attributable to the CRISPR technology. This technology is advancing rapidly, at times surpassing regulatory efforts. Several nations have setup accelerated approval procedures for products based on CRISPR as this technique enables researchers to perform transition from the lab to the field and the shelf considerable times faster when compared to genetic-modification strategies practiced previously.

Pod shatter is a syndrome that poses a constant impediment to canola farmers. Under windy and rainy conditions, pod shatter occurs wherein the pods break open before maturity and dump the seeds on the field where it is unable for machines to pick them up. This results in 10%–40% yield loss per acre. The biotechnology company Cibus has bred canola plants devoid of pod shatter using the gene editing technology and this product is expected to enter into the canola market (Linden, 2023).

Another canola variety resistant to the fungus, white mold or Sclerotinia has been developed by Cibus. Sclerotinia destroys plants and necessitates farmers to turn to fungicides that result in water pollution. This trait conferring Sclerotinia resistance is very valuable as there is no other technology capable of providing resistance to this fungus. Also, biotech companies can stack plant traits such that the market can sell seeds with multiple traits such as herbicide tolerance, disabled pod shatter characteristics and disease resistance. Canola and soybean varieties with herbicide tolerance traits have been generated by Cibus. Cibus has partnered with the fourth biggest provider of soybean seed, Group Don Mario to sell soybean seeds having herbicide tolerance. Cibus along with its soybean and canola partners expects an 80-million-acre market (28 million acres canola and 50 million acres soybean) that could eventually generate royalites over $500 million a year.

### Examples of successful research on gene editing in crops

Several plants have been gene edited for augmenting crop quality, yield and sustainability (Prado et al., 2024). In Oryza sativa, the gene encoding cytokinin oxidase/dehydrogenase (OsCKX11) was knocked out; this resulted in a notable increase in tiller, branch and grain number when compared to the wild type (Zhang et al., 2021). In wheat and cucumber plants, the endogenous genes encoding host factors have been edited, resulting in virus resistance (Robertson et al., 2022). The E1 gene of soybean was knocked out, which led to development of plants having early flowering when subjected to long-day conditions (Han et al., 2019). When the MaACO1 gene was modified in banana plants, it delayed fruit ripening from 3 weeks to 80 days, hence extending fruit shelf life (Hu et al., 2021). Modification of genes in the α-kafirin family of sorghum resulted in augmented protein quality and digestibility (Li et al., 2018a). CRISPR-based engineering of genes in the α-gliadin family of wheat resulted in grains with low gluten content (Sánchez-León et al., 2018). In potatoes, knockout of the gene encoding sterol side chain reductase 2 led to a decrease in toxic steroidal glycoalkaloids (Zheng et al., 2021).

The CRISPR technology has been widely employed for generating gene edited tomato varieties. Resistance to the weed Phelipanche aegyptiaca, the root parasite, was achieved in tomatoes when the MAX-1 gene was mutated by gene editing (Bari et al., 2021). Also, when SlVPE5 and SlINVINH1 genes were simultaneously knocked out, it increased glucose and fructose levels, thus enhancing fruit sweetness (Wang et al., 2021). Tomato plants mutated in the ENO gene developed larger and multilocular fruits (Yuste-Lisbona et al., 2020). The SlHKT1; 2 allele conferring salt tolerance was knocked-in, which led to plants tolerant to germination under salt conditions as high as 100 mM NaCl (Yuste-Lisbona et al., 2020). Multiple or single mutants of the genes Blc, LCY-E, LCY-B2 and SGR1 obtained through gene editing resulted in increased lycopene content in the fruits (Li et al., 2018b). SlAMS gene mutations resulted in male sterility which decreased the cost of production of F1 seeds (Bao et al., 2022).

The gene editing tool, CRISPR/Cas9 has also been developed for improvement of bananas. By editing the gene for lycopene epsilon-cyclase in the banana Grand Naine Cavendish cultivar, the β-carotene content was enhanced (Kaur et al., 2020). These edited bananas showed increased accretion of β-carotene content by as high as 6-fold within the fruit pulp in comparison to that of unedited plants. In addition to this, genome editing of gibberellin 20ox2 in the Gros Michel banana cultivar resulted in plants with decreased height (Shao et al., 2020). When the gene encoding aminocyclopropane-1-carboxylase oxidase was edited in the banana variety, Musa acuminata (AAA group, cv. Brazilian), plants showed traits such as reduced height and deferred ripening that enhanced shelf life (Hu et al., 2021).

### Off-target effects during genome editing

However, despite the rapid progress in the genome editing technology and its positive outcomes, this system faces inherent drawbacks such as non-specific off-target effects (Kadam et al., 2018)). The basic tenet of genome editing is endonuclease engineering to introduce targeted DSBs in order to insert desired alterations into the genome of any given organism. Such endonucleases have been known to result in precise DSBs at the respective targeted loci. Subsequently, these DSBs are repaired via homology-directed repair (HDR) or by non-homologous end-joining (NHEJ) (Rouet et al., 1994). Whereas the HDR is a mechanism entailing high precision, the NHEJ is a comparatively error-prone process. Such advanced GE strategies are intended to generate DSBs at only the requisite sequences guided and targeted by the sgRNAs without introducing any changes in the rest of genome.

Nevertheless, experimental results show that these endonucleases can act on non-specific and non-selective regions within the genomic DNA typically deemed as “off-target” effects (Wolt et al., 2016; Klein et al., 2018; Zhang et al., 2018b; Zhang et al., 2018a)). Many cleavages rather than single, precise cut within the host genome increase the likelihood of chromosomal rearrangements and instability. These include inversion and deletion of DNA fragments within the same chromosome or DNA segment translocation if the cleavage occurs in two distinct chromosomes. Such unbridled and random off-target activities pose challenges to the meaningful investigation of mutations and may result in debilitation of physiological functions (Pattanayak et al., 2011; Sander et al., 2013; Cho et al., 2014; Guilinger et al., 2014; Tsai et al., 2015).

Methods used for detecting potential off-target effects focus on the screening of only a small number of genomic loci that are predicted as likely off-targets using online tools rather than non-discriminatory approaches involving the whole genome (Zhao and Wolt, 2017)). Off-target identification within the genome of edited crops is imperative to preclude their detrimental effects. While the first generation T0 genome edited plants maybe back-crossed to generate null segregates to obviate undesirable and unintended mutations, the breeding involved in backcrossing will extend the requisite time before it can reach the farmers. Hence, the development of potent measures to detect off-targets and research concerning their associated effects would be favorable to the generation of genome edited plant varieties towards agricultural applications in the near future.

### Off-target identification

Despite the astounding success of genome editing technologies and the low rate of DSB occurrence, the principal challenge the needs to be addressed is the validation and quantification of off-target DSB incidents. Moreover, the efficiency of the DSB repair mechanism (HDR or NHEJ) differs significantly based on the type of cell and the state of the cell cycle (Cox et al., 2015). Presently, the most sensitive and precise method to quantify endonuclease-generated mutagenic effects is contingent on high-throughput next-generation sequencing (NGS) technology. For analysis using NGS, the initial step is the amplification of the concerned genomic regions. Deep sequencing having extensive coverage enables the detection of unfavorable mutations (Tsai and Joung, 2016). Particularly, alignment of sequences of the NGS sequencing data by the use of bioinformatic tools can be employed to identify un-intended deletion or insertion mutations that are commonly created in the NHEJ-associated repair pathway. The NGS technology has grown phenomenally in terms of sequence elucidation of huge data. Nevertheless, there is still lack of the requisite sensitivity for direct detection of rare mutations at sites that are off-target, attributable to the high error-rates in the NGS methodology (over 1 in 1000). With such inherently elevated error-rates in sequencing, it is a challenge to discriminate between the artifacts introduced by NGS and the off-target mutations. This degree of error is inadequate to detect cells harboring genomic sequences occurring in the range of several million bases.

The novel strategy of single base editing enables the conversion of a single nucleotide base into another in a programmed and irreversible fashion without the involvement of DSBs (Komor et al., 2016; Gaudelli et al., 2017). In particular, errors occurring in PCR amplification prior to sequencing and the errors introduced in amplifying single molecules or sequencing through synthesis have to be curtailed. Furthermore, improved sensitivity in identifying off-target sites will be favorable. Some of the errors are onerous to identify. For example, the mutagenic effects could be random and therefore rigorous design as well as sequencing of the same sample in many replicates could be used to conclude on the quality consensus sequence. Notwithstanding, systemically accrued or non-random errors like off-target mutations are hard to disprove through this process. In addition, replication errors or mutations occurring due to the activity of thermostable polymerases during the early rounds of the standard PCR amplification reactions are a challenge to distinguish from the original mutations generated due to endonuclease activity. Alternately, strategies using non-exponential amplification could be employed to identify the undesirable mutation.

Genetic information redundancy and the number of homologous sites within the chromosome is a notable factor in the design of sgRNAs. For plant systems, a web tool called “CRISPR-P″ has been formulated to design sgRNAs (Lei Y, 2014). Besides, the choice of the cleaving endonuclease such as fully active Cas9, nickase Cas9, dCas9, C2c2/Cas13a, C2c1, Cpf1 is critical in genome editing. The most characterized endonucleases for genome editing are Cas9 and Cpf1 which possess many distinct properties including the requirement of PAM, PAM-distal region within the gRNA as well as sgRNA-processing capability. Gene expression levels and the stability of the endonucleases, both augment enzyme concentration. High concentrations result in increase of off-target activity by interaction with non-specific sites within the genome. Customized endonucleases can be transiently expressed from plasmids and in brief time periods to decrease the off-target activity. Further, viral vectors that do not integrate with the genome are preferable to minimize off-target activities (Lombardo et al., 2007; Rahman et al., 2013; Holkers et al., 2014). Moreover, target sequence localization inside the genome affects its accessibility and the efficacy and specificity of enzyme activity (Pattanayak et al., 2013). The principal reason for differences between *in vivo* experimental data and *in silico* analyses is target sequence accessibility (Guilinger et al., 2014). Besides, epigenetic modifications influence locus accessibility and the resulting enzyme activity.

In instances where the whole genome of a given crop plant is known, gRNA selection with decreased off-target candidates is achievable by means of online tools. Nevertheless, for several crops, the genome has not been fully characterized and different cultivars within the same species may exhibit polymorphism at different loci. In contrast to Cas9, the Cas9-paired nickases have been found to generate precise, specific on-target mutations while suppressing the off-target mutations (Mikami et al., 2016). The modified spCas9 (nCas9) having nickase activity causes single-stranded breaks rather than cleaving two strands at the target site, thus minimizing off-targets (Ran et al., 2013; Wang et al., 2015; Frock et al., 2015)). Another strategy to decrease non-specific cuts is the fusion of Fok1 nuclease domain to the inactivated Cas9 protein domain (dCas9 (Tsai et al., 2014); wherein Fok1 action is dependent on dimerization. DSBs generated in this manner require the interaction of two sgRNAs to the target sequence in a predefined process.

### Examples of off-target investigations in crops

Use of the CRISPR/Cas9 technology in *Triticum aestivum* (common wheat) and *Oryza sativa* (rice) revealed that deletion events are introduced because of NHEJ repair of the 5′overhangs that result from Cas9-elicited DNA breaks (Shan et al., 2013; Zhang et al., 2014);. Two copies of the gene HvPM19 were targeted in *Brassica oleracea* and *Hordeum vulgare* using Cas9-based editing which was found to induce mutations in 10% and 23% of the lines respectively (Lawrenson et al., 2015);

As per Basu et al., 2023, stable Cas9-elicited mutations were transmitted to T2 plants in both species. While there occurred no less than one mismatch between the sgRNA used and the non-target sequences, in both plants off-target activity was identified. A *H. vulgare* plant free of the transgene showed mutations in both target and non-target HvPM19 alleles.

Gurel et al. (2023) used the rice system to target GRAIN SIZE 3 (OsGS3) and EPIDERMAL PATTERN ING FACTOR LIKE 9 (OsEPFL9) genes respectively with ATTC and GTTG protospacer adjacent motifs. They used Aac and Aa1.2, two *Alicyclobacillus acidoterrestris* scaffolds to significantly augment the frequency of highly targeted mutagenesis. Stable transformed T0 rice plants were subjected to whole-genome sequencing (WGS) to identify off-target mutations. This revealed the occurrence of background mutations in the noncoding and coding regions and there was no display of any off-target activity of the sgRNA in the edited rice genomes. In the T1 generation, there occurred Mendelian segregation of indel (insertion and deletion) mutations. Both the Aac and Aa1.2 scaffolds enabled heritable and precise genome editing substantiating the success of using the CRISPR/AaCas12b system towards crop improvement.

Sretenovic et al. (2023) assessed the off-target activity of the BASE EDITOR8e (ABE8e) and its high-fidelity form, ABE8e-HF in *Solanum lycopersicum* (tomato) at 2 distinct target sites in tomato protoplasts and in stable T0 lines. The ABE8e version showed greater on-target efficacy compared to ABE8e-HF in protoplasts due to which the ABE8e was chosen for off-target identification in T0 lines. Whole genome sequencing (WGS) was conducted in wild-type tomato plants, T0 lines expressing GFP, T0 control lines with ABE8e-no-gRNA as well as the edited T0 lines. No off-target edits attributable to the gRNA were detected at both genomic and transcriptomic levels.

In tomato, equivalent editing efficiencies (8.8% and 7.3% respectively) were observed for CRISPR/Cas9 and TALEN editing mechanisms upon the use of geminiviral replicons for overexpression of a critical transcription factor Anthocyanin mutant 1 (ANT1) regulating the anthocyanin biosynthesis pathway. Insertions were introduced at the targeted site, and the analogous purple phenotypes elicited by both editing systems showed heritability and there was no evidence of off-target edits (Čermák et al., 2015).

Fan et al. (2024) compared TadA-8e-derived Cytosine base editors (CBEs) in tomato and rice cells. From this, they identified TadC BEd_V106W, TadCBEa and TadCBEd as efficient CBEs having high purity and a limited editing window. TadDE, a dual base editor performs simultaneous A-G and C-T editing. TadDE- and TadCBEa-mediated multiplex base editing was shown in transgenic rice with no detection of off-target effects as proven by transcriptome and whole genome sequencing. This indicated the high specificity of this editing system. Herbicide resistance alleles were introduced in OsALS using TadDE which created synonymous mutations in the gene OsSPL14 that resulted in resistance to OsMIR156-enabled degradation. This investigation showed that CBEs derived from TadA-8e as well as a dual base editor are important in plant genome editing.

### Long term effects and other concerns

The long-term effects of genome editing must be evaluated as well. Genome-edited crops may outcompete native plant species or have other unintended ecological consequences. These could include the potential impact on natural ecosystems, for example, possible gene flow to wild or non-target plants, an impact on nontarget organisms, and possible implications for beneficial insects. Alterations in gene expression could change any of these interactions.

In terms of food crops, regulations must include the possibility of accidental unexpected outcomes. The possible existence of allergens or toxins that could pose risk to human health should be investigated. For example, a genome edited potato produced more acrylamide when cooked (Tussipkan and Manabayeva, 2021). These are issues that must be addressed with classical breeding technologies as well. Ways to mitigate these risks would be the use of bioinformatic tools and computational methods to look for truncated genes or altered biosynthesis pathways. In addition to this, the use of current risk assessment tools such as toxicological assays and sensory evaluations should be maintained (Movahedi et al., 2023).

Finally, the development and deployment of genome-edited crops could have significant social and economic impacts, both positive and negative. Thus, post-market analyses need to be performed.

### Regulation of genome editing in North America

The commercialization of CRISPR-edited crops faces many hurdles, including regulation, public acceptance, and whether such crops are classified as GMOs or non-GMOs. The current global regulatory landscape for CRISPR-edited plants is patchy and many of the world’s less-developed countries have not yet devised regulatory systems for assessing CRISPR-edited plants (Ahmad et al., 2021).

In the case of genome editing, small genetic changes in plants such as point mutations should be easier to navigate through regulatory bodies. Regardless, there is some concern among the public sector that societal acceptance and regulatory clarity might remain as sticking points (Gleim et al., 2020).

The regulatory landscape for genome edited crops continues to evolve (Keiper and Atanassova, 2022). Regulation of genome editing is itself challenging, as in some instances, gene insertions are made in a manner that is comparable to the generation of transgenic crops, known as SDN-3 (or site-directed nuclease level 3) events. In other instances, however, small deletions or point mutations can be included that render the plant indistinguishable from plants generated using conventional breeding methods (site-directed nuclease-1, or SDN-1 events) (Jenkins et al., 2021). As such, a growing number of regulatory agencies use a case-by-case approach, with SDN-1 events requiring the least oversight and those plants created using SDN-3 events requiring a regulatory framework that more closely resembles the one used for transgenic plants. In the latter case, an approach that takes into account the safety/novelty of the trait, intended use for the crop (human food, animal feed), and potential for environmental risks in the field must take place. The culmination of these regulatory processes results in a significant investment for developers of transgenic technologies, with cost to commercialization reaching USD $136 million over a 13-year timeline. Such exorbitant costs and timelines for transgenic crops have been a major deterrent for small businesses and the public sector, and as a result, the commodity crops soybean, cotton and maize dominate the commercialised GMO landscape. These three crop types express traits that are useful to farmers, such as insect and herbicide resistance (National Academies of Sciences, 2016; Whelan et al., 2020). Regulatory agencies in countries such as Argentina, Australia, Brazil, Canada, India, Japan, Philippines, and the United States have implemented that genome editing to create point mutations may not fall within the scope of transgenic plant regulation (Entine et al., 2021; Mallapaty, 2022). These countries will also take into account genome editing methodology (CRISPR, TALEN, etc) (Touzdjian Pinheiro Kohlrausch Távora et al., 2022), and whether the type of product more resembles one that could be achieved by conventional breeding or mutagenesis, rather than transgenesis. The European Union and other Latin American countries such as Peru, Bolivia and Mexico have outright banned the growth and commercialization of genome edited crops (Zarate et al., 2023).

Over 3 dozen different genome edited plants have been released for growth throughout the US and Canada. Examples include a non browning mushroom, ‘waxy’ corn composed of amylopectin, and “Sicilian Rouge High GABA’ a variety of tomato that produces an amino acid that battles chronic disease. Similarly, in Brazil, a genome edited form of sugarcane that contains higher sugar content, and in Argentina, a genome edited potato with reduced browning traits (Touzdjian Pinheiro Kohlrausch Távora et al., 2022) have been released. Many more examples include resistance to pests and abiotic stresses, such as extreme temperatures and drought. Genome edited crops can also increase genetic diversity to improve current domestic crops, and even enable wild crops to become domesticated (Mamrutha et al., 2024; Van Tassel et al., 2020). Another study described in (Alvarez et al., 2021), presents three case studies: the Arctic^®^ apple, the Pinkglow pineapple and the SunUp/Rainbow papaya in terms of genetic engineering regulations. The detailed genome editing regulations applicable in the US have been reported by Wolt and Wolf (2018).

Genetic diversity is the primary factor for trait improvement in crops. Genetic variations within the gene pool are the major requirement for developing novel plant varieties. Since the early twentieth century, various tools have been introduced for crop breeding. While these crops may be released and available for commercial sale in certain countries which already possess robust frameworks that are conducive for the commercialization of transgenic plants, regulatory frameworks vary across different regions, such as the European Union and many of her trading partners (Mamrutha et al., 2024). The result of this disharmony is a patchwork of regulations across the globe, which may further confound the advancement of new breeding techniques to low and middle-income countries.

### Obstacles in Europe

While much of the research on gene editing and other technologies previously referred to as “New Plant Breeding Techniques” (NPBTs) had been developed in European research centers and universities, there have been several regulatory obstacles to the marketing of these innovations.

In general, European regulations take a process-based approach rather than a product-based one so even if there is no difference between a gene-edited seed and its original source, the EU would still regulate on the process. This regulatory bias evolved, in part, from the EU’s reliance over the last 2 decades on the precautionary principle which takes a hazard-based approach to new technologies rather than the more risk-based (exposure-focused) approach in US regulatory bodies.

Opposition to NPBTs grew out of a conflict within the organic food community. More than a decade ago, there was an open debate about whether certain gene-edited seeds and certain seed breeding technologies could be allowed under the organic food regime (Andersen et al., 2015). At the time, developments in reducing potato blight without pesticides via cisgenesis had attracted a considerable amount of attention as organic potato yields were suffering (Lammerts van Bueren et al., 2008).

The debate within the organic food community was quite noisy and public, but by 2016, the left-wing of the movement within IFOAM, that had identified seed breeding with industry and patent control, won the argument (Group, 2015). NPBTs would not be allowed under the organic farming regime.

Around this time, the agroecology and peasant farming wings were gaining in influence and funding and the green political policy moved to take a hard line on all novel seed breeding techniques (Zaruk, 2018). Far left, anti-industry activist groups like Corporate Europe Observatory (which employ no agronomists or scientists) were taking the policy lead, declaring that any of the seed breeding techniques were merely GMO 2.0 that needed to fall under the 2001 GMO Directive (Observatory, 2016).

The European Commission, DG Santé, recognizing the conflicts among stakeholders, was delaying publishing a working paper on the matter or even defining what constituted a new plant breeding technique. There were at least seven breeding technologies that were bundled under the category “NPBTs”: Site-Directed Nucleases like CRISPR; Mutagenesis; Cisgenesis; DNA methylation; Grafting (non-GM scion on GM rootstock); Reverse breeding; and Agro-infiltration (Agostino, 2017). This bundling created confusion and discrimination in how the new techniques worked and the benefits they could provide to agriculture. This was frustrating the research community that was watching the innovative opportunities being developed without any clarity on European regulations.

In 2014, the European Parliament passed a resolution demanding regulation on plant breeding, namely NPBTs (Parliament, 2014). The European Commission never acted to regulate then, merely holding an endless series of meetings and consultations with no conclusions (Commission, 2023). Rather, in the absence of any clear policy, an NBPT complaint was forced on the European Court of Justice. A consortium of nine French peasant farming groups and NGOs had filed a case with the courts aimed at the potential risks from certain mutagenized herbicide-resistant seeds and in July 2018, the court ruled in their favor (Union, 2018). Any new plant breeding techniques would have to fall under the outdated and repressive 2001 GMO Directive making their marketing within the European Union impractical (Union, 2001). The global scientific community condemned this decision (Niiler, 2010) but the European court had no choice in the matter (and did not have the technical competence to decide on such technologies) given there were no other legal frameworks to base their decision.

Any gene editing, no matter how benign, would fall under the same regulatory restrictions as transgenic interventions meaning it would be very expensive and time-consuming to bring innovations onto the market. Even if a gene-edited seed is indistinguishable from the native seed, it would still be considered as genetically modified (a GMO).

This European Court decision gave birth to an ecomodernist movement among the young plant biologist community. A group of students started a European Citizen Initiative to revise the 2001 GMO Directive called “Grow Scientific Progress” (Initiative, 2021). Other groups, like Give Genes a Chance, started using NGO campaign tactics against the green activist positions (Researchers, 2023) European researchers had mobilized.

By the time of the Farm2Fork initiative, in 2020, European policymakers were giving gene editing a rethink. To integrate agriculture within the Green Deal climate objectives, certain actors in the European Commission within Commissioner Timmermans’ DG felt that serious cuts needed to be made to agricultural inputs to reduce farm-based CO2 emissions (Commission, 2022). But in calling for an expansion in land dedicated to organic farming practices while radically cutting pesticide and fertilizer use, Commission advisers, particularly in the EU’s Joint Research Centre, made it clear that yields would fall dramatically (Repository, 2021). A 2022 impact assessment by the Dutch University of Wageningen concluded similar yield reductions (Research, 2022). Rather than watering down the original Farm2Fork objectives, the European regulators decided to take another look at innovative seed solutions that could lift yields to compensate for the forecasted declines due to the climate-driven restrictions.

In 2021, the European Commission published a study on New Genomic Techniques (NGTs), changing the name perhaps as a way to move on from their failure to act on NPBTs (Safety, 2021). NGTs were defined “as techniques capable to change the genetic material of an organism and that have emerged or have been developed since 2001, when the existing GMO legislation was adopted” (Ibid). The Commission study though raised more issues than it had resolved, concluding that “recent developments in biotechnology, combined with the ambiguity of definitions, still impede the interpretation of some concepts” (Katsarova, 2024). The need for regulation was imperative.

In 2022, EFSA published the criteria for a risk assessment on certain NGTs (EFSA GMO Panel, 2022) and in May 2023, the European Commission published its Impact Assessment on plant NGTs (Union, 2023a).

Following this, the European Commission tabled a proposal for a regulation on plant NGTs in July 2023 (Union, 2023b) that was then passed by the European Council and Parliament in the first readings in April 2024. There was however an amendment passed in the Parliament stipulating that certain gene-edited seeds could not be patented (Parliament, 2024).

This was a clear compromise to the environmental activist community that had always campaigned against patenting seeds, even though such a ban would go against international trade agreements and the European Patent Convention. The European Parliament asked for a report by June 2025 to assess the effects of patents on access to seeds. In any case, the proposed patent restrictions will be assessed in trialogue (between the three main European legislative branches) prior to a second reading.

Big Ag has dominated the GMO approvals because of the cost and time needed to bring a trait onto the market. Small and medium-sized enterprises (SMEs) are unable to afford to meet the regulatory demands, often extending over a decade (and often not approved at the end). Large multinationals have deeper pockets to survive the process and often work with the smaller companies or take them over in order to bring the seeds to market. To recoup expenses, biotech companies need to focus on high volume cash crops like soy, maize, canola and cotton. The observations of (Whelan et al., 2020), that the GMO market is dominated by multinationals and a limited number of crops can be explained by the demanding regulatory process. This can also be seen in the pharmaceutical approval process that favors large companies and drugs for more common diseases (note the GM approval process mirrored the pharmaceutical approach).

For certain NBTs, a lower regulatory environment would certainly support SMEs and academic research, as well as a democratization of biotech that would promote solutions to less common crop issues (like the Ugandan lab that found a solution to the local banana wilt crisis). But regulations are tightened when there is uncertainty and risk so there is question if NBTs could survive the regulatory honeymoon (Whelan et al., 2020) seems to assume. NGOs can raise fear and uncertainty (i.e., GMO 2.0), food companies could reject technologies without sufficient controls (like how McDonald’s refused to use the Simplot Innate potato for fear of a market backlash). Regulations tend to reflect cultural values (like a demand for traditional or natural foods) so Whelan’s focus on an ag-industry concentrated economy in Argentina is quite different from the European perspective.

### Consumer acceptance and governance in the United States

An interesting article by Bearth et al. (2024), compared consumer acceptance of three genome edited crops in the United States vs. Switzerland, two countries that widely differ regarding genome edited crop regulation. Using an anonymous online survey of over twelve hundred participants, the study explored what consumers from these two countries thought about three specific applications of NGTs in plant breeding (blight-resistant potato, gluten-free wheat, and cold-resistant soybean). Interestingly, participants from both countries expressed positive feelings regarding the three applications, one quarter of the participants expressed negative emotions, and the remaining participants were neutral to these applications of genome edited crops. The study mentions that regulations or outright bans in one country can impact public perception in other countries. We have seen such events this year in the pull back on release of GM crop Golden Rice in the Philippines and the impact this has had on countries such as Bangladesh and India. It has also been suggested that labeling of genome edited foods could have a negative effect on consumer acceptance, while the availability of foods with consumer benefits, such as improved nutrient content, may shift consumer perception into greater acceptance. Another factor that has influenced acceptance of genome editing has been the viewpoint of some that crops developed in this fashion are in some consumers perception considered to be tampering with nature and “unnatural.” In the United States, fewer negative attitudes toward GM products were found amongst those with higher scientific knowledge scores.

In the United States, the regulation of NGTs is primarily governed by the Coordinated Framework for Regulation of Biotechnology from 1986 (51 Fed. Reg. 23, 302), administered by three federal agencies: the Environmental Protection Agency (EPA), the Food and Drug Administration (FDA), and the United States Department of Agriculture (USDA) (Turnbull et al., 2021; National Academies of Sciences, 2016; Waltz, 2016). This Coordinated Framework is based on the regulation of its product, not the process. In 2020, the USDA also released SECURE (Sustainable, Ecological, Consistent, Uniform, Responsible, Efficient) rules for biotech crops. In the past, the Animal and Plant Health Inspection Service (APHIS) considered all plants generated by means of genetic engineering to be plant pests under its 7 CFR Part 340 regulations as the genetic engineering approaches used at the time (during the 1990s) generally used genetic material sourced from plant pests to change the genetic properties of target plants. Therefore, the plants were subject to review and approval by the APHIS before commercialization. In 2021, an NGO group challenged important provisions of the USDA regulations determining the way to market new GE plants including those generated by the use of gene editing tools such as CRISPR. The plaintiffs contended that the exemption of several GE crops from pre-market assessment and permissions violated the APA (Administrative Procedure Act). As per the ruling of 2 December 2024, the court determined that the USDA did not articulate a reasonable basis for certain features of the final rule as necessitated by the APA. To remedy this situation, the court rescinded the rule on a proposed basis. Given the significance of gene editing to the innovation of the food and agricultural sectors, this decision is an important one that has major implications for these sectors in the future. The decision of the court stands as a setback in attempts regarding multiple administrations to follow a risk-based strategy to GE crop regulation that focuses on plant characteristics themselves instead of the process employed to develop them. Nearly 100 new GE crops inclusive of several gene-edited crops have obtained acceptance of exemption determinations ever since the final rule was issued in 2020 (Bruce, 2024).

Currently, regulations imposed by the APHIS exempt GE crops incorporating modifications from regulations, provided the same modifications could have been generated via conventional breeding. Therefore, a given GE crop is not considered as a noxious weed subject to regulations unless its non-GE equivalent is also a noxious weed. However, newly generated GE crops now face more uncertain and likely more formidable regulatory path to the market with apparent implications for agricultural and food sector innovations until the APHIS is able to answer the concern of the courts whether by the appeal of the rule or by the restatement of this final rule in a form that passes gather under the APA.

Canada’s GM regulatory framework is product-based, and similarly to the US, does not consider the processes used to create the plants from which the products come (Barrangou, 2020). However, Canada is unique in that all novel traits in plants, whether they are developed by conventional breeding, traditional mutagenesis or targeted mutagenesis are assessed under the same risk assessment regulations by the Canadian Food Inspection Agency (CFIA). Canada’s assessment of novel plant risks is stringently science-based, focusing on toxicity, off-target impacts, and product allergenicity (Ahmad et al., 2021).

The US and Canada have a long history associated with GM crops; both countries were among the first to make concrete decisions upon the regulatory status of several new plant breeding innovations (Ewa et al., 2022; Gleim et al., 2020; ISAAA, 2021). As a result, the US leads biotech crop planting at 71.5 million hectares, and over 90% of US corn, cotton, and soybeans are produced using transgenic varieties (ISAAA, 2019). Similarly, Canada has been assessing GM crops for over 20 years, with over 140 transgenic crops approved for sale. Brazil and Argentina are also top producers of GM crops; other Latin American countries which approve GM crops are Chile, Colombia, Guatemala, Honduras and Paraguay, on the other hand, Ecuador, Venezuela, and Peru do not permit commercial cultivation of GM crops within their lands (Turnbull et al., 2021). In December 2020, Mexico issued a ban of GMO corn.

For United States and Canadian consumers, GM crops make food more affordable (McFadden and Smyth, 2019; Brossard and Nisbet, 2007; Besley and Shanahan, 2005). US farmers, the primary consumers of GM seeds, felt confident that GM crops had undergone thorough scientific risk assessment by the authorities and did not perceive human or animal health to be issues (Madsen et al., 2003). In 1930, American households spent 21% of disposable income on food, while this figure fell to 5.7% in 2012 (Goodwin et al., 2015). One of the contributing factors has been the introduction of genetically engineered crops: comparable figures for countries that have rejected genetically engineered crops, such as Germany and France are 10% and 13%, respectively (McFadden and Smyth, 2019). More than 56% of respondents among US consumers were willing to buy and consume both GM and GE food (Shew et al., 2018). Canadian consumers were unlikely to buy a GM product at a higher price compared to a non-GM one, even if it had a better nutritional profile (Macall et al., 2021). Thirty nine percent (N = 506) were quite price-sensitive as they indicated that they would likely and very likely buy a nutritionally enhanced GM product if the price were the same as a non GMO product (Macall et al., 2021). This is an interesting result, because it would suggest that what really drives consumer’s purchasing decisions is not the production method but the price of the product.

It must be stated that consumer acceptance is affected by reliability of the information received and whether it is based on fact, opinion, or a deliberate intent to mislead (Caradus, 2023). Disinformation is false or misleading content purposefully created with an intent to deceive and cause harm and is motivated by the desire to influence, profit, or engender. Disinformation contributes to an erosion of social cohesion. In a voluntary survey about the use of New Breeding Technologies in crops, it was found that the dominating factor (38% of respondents) influencing attitudes was “public confusion about food safety and health risks” of these new technologies (Friedrichs et al., 2022).

The fact that there is no international consensus about regulation of genome edited plants has cumulated into tensions even within the agbiotech industry itself (Selfa et al., 2021). The results of literature analysis show that the public in Europe and North America is more familiar with the notion of genome editing and genetically modified organisms than the public in other world regions (Ewa et al., 2022). GM crops themselves remain a concern for different interest groups, along the lines of vaccinations or climate change. Consumer attitudes toward it widely differ from strong criticism to strong approval. For example, several points of conflict frequently take place between different stakeholders. While scientists often describe genome editing as identical in terms of point mutations compared to conventional breeding, and thus downplay its link to GM crops and associated negative connotations, they also recognize that sensible regulations are required to foster public trust this new technology. Environmentalists, on the other hand, might make the effort to incorporate GMO negatively into genome editing, and even require labeling, which would likely lessen consumer confidence, as some consider labeling a red flag or a warning. Meanwhile, large corporations already practice self regulation and have set a number of checks and balances in place to ensure that food products are safe. These stakeholders would thus be comfortable with incorporating genome edited crops into the current infrastructure. Small corporations and public institutions thus fear that those who mistrust large multinationals may urge for over-regulation in the future. Small companies fear that loss of consumer trust will doom the genome edited industry, rather than level the playing field and open opportunities for others. However, lots of regulations will make it difficult for small entities to go through approval process. Finally, in the case of the Global South, the fear of another fiasco with agricultural biotechnology is of great concern, as products of GMOs have largely been withheld from them. Genome editing could offer the promise of greater access to agriculture technologies in general.

## Conclusion

This review has sought to describe both the extraordinary commercial potential of genome edited crops as well as the regulatory challenges that are associated with their implementation in the world today, with particular emphasis on the Americas and Europe. The discrepancy in regulatory approval strategies between various regions of the Global North will have a profound impact on the Global South, who has the most to gain from newly emerging technologies in plant breeding and agriculture. Regardless, more than 30 countries (many of them low and middle income) have now developed amended regulations surrounding genome edited crops, and even more are in the process of doing so (Groover et al., 2024; Pixley et al., 2022; Dionglay, 2024). Genome editing could inherently help democratize the benefits of science for LMIC, for they are inexpensive to implement and practical for providing useful new traits to orphan crops, for which funding can often be scarce. All of this will bring benefits to smallholder farmers.

Future efforts to unify regulatory standards will be highly influenced by consumer acceptance in locations such as the US versus the EU. As a result, while genome editing has much to offer, the road to its attainment as a fundamental method to improve agricultural outcomes will remain rocky for time to come.
